# Estimating the effect of self-protection on transmission dynamics of SARS-CoV-2 in Germany in 2021: a modelling study

**DOI:** 10.1186/s12889-026-27264-w

**Published:** 2026-04-14

**Authors:** Marvin Schulte, Neele Leithäuser, Jan Mohring

**Affiliations:** 1 Fraunhofer Institute for Industrial Mathematics, Fraunhofer-Platz 1, Kaiserslautern, 67663 Germany; 2 Fachbereich Mathematik, Rheinland-Pfälzische Technische Universität Kaiserslautern-Landau, Gottlieb-Daimler-Str. 48, Kaiserslautern, 67663 Germany

**Keywords:** Behavioral-epidemiological model, Adaptive behavior, Compartmental model, Infectious disease dynamics, Epidemiology, COVID-19, Parameter estimation

## Abstract

**Background:**

During the COVID-19 pandemic, German states implemented non-pharmaceutical interventions, while individuals also adopted self-initiated protective behavior. Most epidemiological studies tend to focus on one of these aspects, but in reality, both factors influence transmission dynamics simultaneously. In this study, we investigate the effect of self-protection and NPIs on the transmission dynamics of SARS-CoV-2 in Germany during 2021 and identify the corresponding model parameters based on publicly available data.

**Methods:**

We present a unified mathematical model that integrates both self-initiated protective behavior and mandated policies. By using infection and intensive care unit data from four German states, we identify all behavioral and some viral parameters, while some are set according to literature values. Since the data alone do not reveal the cause of reduction, we use the different functional structures of self-protection and non-pharmaceutical interventions to determine their respective influence. Based on these parameters, we conduct counterfactual simulations, modeling the absence of one of the mechanisms, respectively, while assuming that the other mechanism is left unchanged.

**Results:**

Our findings indicate that both mechanisms substantially reduced transmission. Self-protection reduced the transmission less than mandated policies most of the time, but provided between 67.6 (± 6.9) % and 81.9 (± 2.1) % further reduction of the transmission rate at highest reported values of intensive care unit occupancy. Through counterfactual simulations, we demonstrate that the absence of policies or self-protection would have resulted in higher case numbers or the need for stronger adaptations.

**Conclusion:**

The results emphasize the crucial role of self-protection in addition to mandated policies in controlling the spread of the virus. Our research highlights the importance of incorporating self-protective behavior and mandated policies jointly in epidemiological models, which are used for policy evaluation.

## Background

The COVID-19 pandemic has significantly impacted life worldwide, resulting in millions of deaths and hundreds of millions of infections [1]. In response, governments implemented non-pharmaceutical interventions (NPIs), while individuals also adopted certain measures on their own due to their risk perception [2]. By 2021, the second year of the pandemic, the population in Germany got used to the new virus. However, a high level of risk perception and a willingness to adapt behavior remained prevalent [2].

Adaptive human behavior in response to the spread of an infectious disease has been investigated in epidemiological models since prior to the COVID-19 pandemic. The topic is commonly referred to as behavioral epidemiology (of infectious diseases) [3]. There are multiple reviews on these mostly theoretical models [4–6]. Approaches to incorporate adaptive behavior exist for compartmental models [7], network-based models [8], as well as individual or agent-based models [9]. In compartmental models, either additional awareness compartments are introduced [7] or the transmission rate is modified to a non-constant one [10]. Game-theoretic approaches [11, 12] determine behavioral response to an epidemic based on utility functions. Reluga [13] and Li et al. [14] formulate this as an differential game and differentiate between individual and population-average/public social distancing investment, which can be related to self-protective and mandated behavior change. We aim to investigate how the interplay between these two aspects unfolded during the COVID-19 pandemic.

As the pandemic progressed, multiple models of adaptive behavior were developed and applied to real data, using parameter estimation [15–18], most of whom are compartmental models with a feedback loop modifying transmission parameters [19]. With the exception of Oveson et al. [18], these studies do not explicitly model mandated interventions. On the other hand, there are modeling studies that aim to quantify the effects of NPIs [20–24]. These studies, especially if they are solely data-driven, often do not incorporate adaptive behavior that would occur in response to the outbreak, independent of the interventions. Studies including both effects are often regression analyses focusing on the first wave of COVID-19 [25, 26], but the behavior during this wave probably differed from subsequent waves [2].

Several studies have found evidence of voluntary self-protection behavior in Germany in addition to mandatory measures. Zozmann et al. [27] did a regression analysis of human mobility data, focusing on the influence of NPIs measured by a stringency index and 7-day incidence as a measure of perceived risk. The stringency index is similar to the Oxford COVID-19 Government Response Tracker [28]. The study focuses mainly on the autumn 2020 wave of COVID-19 in Germany. They find that human mobility changed due to both, policy-induced and self-protective adaptation, and recommend including both mechanisms in epidemiological models. Schulze et al. [2] analyze data from the COSMO study [29], a large serial cross-sectional German study based on a survey that also asks for protective behaviors, as a part of their study. They find a connection between the ICU occupancy and the (voluntary) implementation of protective behaviors. This connection is also found in a study by Dönges et al. [30], based on data from the COSMO study as well. The relation is shown to saturate at a certain level and a functional form of the relation is given that is used in an epidemiological model. However, the parameters of this model of self-protective behavior are not fit to data.

In our study, we extend this work by identifying all behavioral parameters based on infection and ICU data from four German states during the year 2021. By this, we are able to understand which effect the two mechanisms had on the transmission dynamics of COVID-19 in Germany during the simulation period. We use these parameters in counterfactual simulations to see if one mechanism alone would have been sufficient to reduce transmission to sub-critical levels in situations where the combined effects reached this.

The findings of this study will contribute to the understanding of the interactions between self-initiated behavior and policy measures and provide insights for planning future public health strategies.

## Methods

In this section, we present the data used, the transmission model and the parameter identification method.

### Data description and sources

In our study, we focus on two key infection-related metrics: the number of detected COVID-19 cases and the occupancy of intensive care unit (ICU) beds by COVID-19 patients. The data originates from Germany, where we model four different federal states independently. This approach allows us to account for the varying NPIs implemented across different states and enables us to estimate how general our findings are. Both datasets (detected cases [31, 32] and ICU occupancy [33]) are provided by the Robert Koch Institute, Germany’s national public health agency, through their GitHub repositories.

**Fig. 1:**
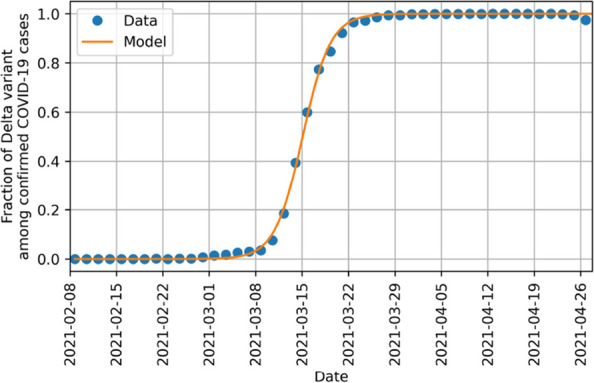
Interpolation of the Delta variant fraction among detected cases in Germany shows a good accordance between the logistic interpolation function and the data

Our analysis focuses on the year 2021, a period when SARS-CoV-2 remained widespread [31, 32], case numbers were extensively reported [34, 35], testing capacities were high [36, 37], and the population’s behavioral response had stabilized following the initial shock [2]. Specifically, we examine data from February 8, 2021, to December 20, 2021, because the infection dynamics during this timeframe were primarily influenced by two major variants of SARS-CoV-2: the Alpha and Delta variants [38]. To reflect the impact of these variants in our model, we categorize the detected cases by variant type. The variant fractions were derived from data provided by the Robert Koch Institute [38]. Given that weekly variant fractions are provided, we employ a logistic growth model, which was shown to describe the variant fractions during transitions [39], to interpolate daily variant fractions. The data and the resulting interpolation function are shown in Fig. 1, demonstrating a strong correlation between the observed data and the interpolation.

In 2021, mass vaccinations were underway in Germany, significantly influencing transmission dynamics [40, 41]. We incorporate vaccination data, also sourced from the Robert Koch Institute [42], into our model.

In addition to this pharmaceutical intervention, various NPIs with differing levels of stringency were enacted across German states throughout 2021. We extracted the dates when NPIs changed from the official regulations of Hesse [43], Lower Saxony [44], Rhineland-Palatinate [45] and Thuringia [46]. We choose these states as we have already used them in a previous study [47] and processed their data. Furthermore, the dynamics and regulations in these states reflect the variety of infection dynamics and regulations across German states.

The data described here is used to fit the parameters of the transmission model.

### Transmission model

To model the transmission of SARS-CoV-2 during 2021, we employ an SEIRS-like compartmental model. We will first outline the basic transmission model, then describe how the transmission rate, including self-protection, is parametrized and finally cover the model extension for two variants. Important features that shaped the transmission dynamics of SARS-CoV-2, including vaccinations [40, 41], underreporting [48–51], seasonality [52, 53], the implementation of NPIs [22, 54] and multiple variants [55, 56] are covered by our model.

#### Basic transmission model

The basic transmission model is an SEIRS-like compartmental model that accounts for a latent and an infectious period. A full derivation of the model from the original equation by Kermack and McKendrick [57] can be found in [58]. Instead of an exponentially distributed latent and infectious period, which leads to the greatest fraction losing infectiousness immediately after getting infectious and can misestimate the infectious peak significantly [59], we use a fixed length of the infectious period with uniform infectiousness. This fixed length is set to the estimated length of the infectious period from epidemiological studies [60]. For classical SEIRS models, the recovery rate is usually set to the inverse of this value such that the expected time of infectiousness equals this length. Nikolaou [59] also shows that more realistic infectiousness distributions yield solutions that are much closer to the fixed length solution than to the classical exponentially distributed infectiousness solution. Our model was already successfully applied to COVID-19 forecasting in the European COVID-19 Forecast Hub [61].

**Fig. 2:**
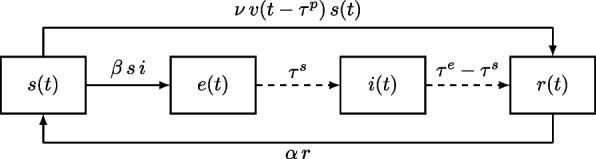
Transitions between the basic compartments with the respective rates and delays. Solid lines correspond to transition with a rate, dashed lines to transitions with a fixed delay

Figure 2 shows the transitions between the basic model compartments. Using fractions, we can write the model as

1 $$\begin{aligned} \frac{\textrm{d}s}{\textrm{d}t}= & -\beta (t)\,s(t)\,i(t)-\nu \,v(t-\tau ^p)\,s(t)+\alpha \,r(t), \end{aligned}$$

2 $$\begin{aligned} \frac{\textrm{d}e}{\textrm{d}t}= & \beta (t)\,s(t)\,i(t)-\beta (t-\tau ^s)\,s(t-\tau ^s)\,i(t-\tau ^s), \end{aligned}$$

3 $$\begin{aligned} \frac{\textrm{d}i}{\textrm{d}t}= & \beta (t-\tau ^s)\,s(t-\tau ^s)\,i(t-\tau ^s)-\beta (t-\tau ^e)\,s(t-\tau ^e)\,i(t-\tau ^e), \end{aligned}$$

4 $$\begin{aligned} \frac{\textrm{d}r}{\textrm{d}t}= & \beta (t-\tau ^e)\,s(t-\tau ^e)\,i(t-\tau ^e)+\nu \,v(t-\tau ^p)\,s(t)-\alpha \,r(t), \end{aligned}$$

with

5 $$\begin{aligned} s(t)+e(t)+i(t)+r(t)=1, \end{aligned}$$

where the term $\nu \,v(t-\tau ^p)\,s(t)$ represents vaccinations which are only protecting after some time $\tau ^p$ and with an effectiveness of $\nu$ as found in studies [62, 63].

**Fig. 3:**
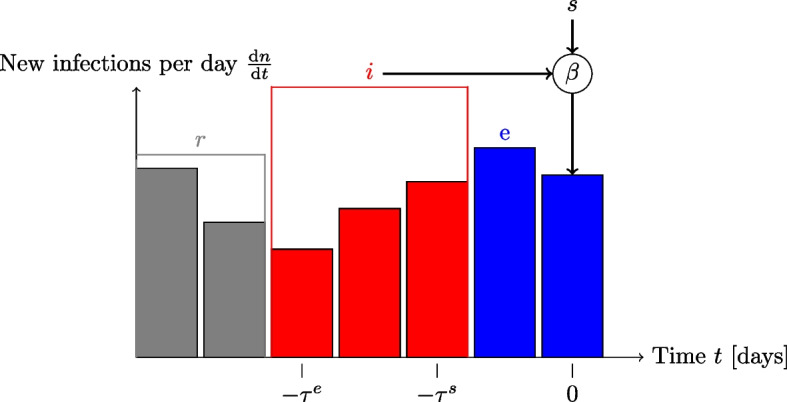
Diagram illustrating the relation between the number of new infections per day and the SEIRS compartments

To compare our model with the reported number of SARS-CoV-2 cases and ICU patients, we rewrite the model in terms of *n*(*t*), which denotes the cumulative number of infections, i.e. the integral over new infections, such that

6 $$\begin{aligned} \frac{\textrm{d}n}{\textrm{d}t}= & \beta (t)\,s(t)\,i(t), \end{aligned}$$

7 $$\begin{aligned} \frac{\textrm{d}p}{\textrm{d}t}= & \beta (t)\,s(t)\,i(t)+\nu \,v(t-\tau ^p)\,s(t)-\alpha \,p(t), \end{aligned}$$

8 $$\begin{aligned} s(t)= & 1-p(t), \end{aligned}$$

9 $$\begin{aligned} i(t)= & n(t-\tau ^s)-n(t-\tau ^e), \end{aligned}$$

where *p*(*t*) denotes the immune (protected) compartment, irrespective of infectiousness. Figure 3 illustrates the relation between the number of new infections and the SEIRS compartments.

The cumulative number of detected cases *d*(*t*) is given by a fraction $\rho (t)$ of all infections with some delay $\tau ^d$, called the detection time, and a fraction $\mu$ of all infections needs ICU treatment after some time $\tau ^{is}$ and leaves ICU after time $\tau ^{ie}-\tau ^{is}$. With *c*(*t*) denoting the actual number of ICU patients, we have

10 $$\begin{aligned} \frac{\textrm{d}d}{\textrm{d}t}= & \rho (t)\,\frac{\textrm{d}n}{\textrm{d}t} (t-\tau ^d), \end{aligned}$$

11 $$\begin{aligned} c(t)= & \mu \left( n(t-\tau ^{is}) - n(t-\tau ^{ie})\right) . \end{aligned}$$

**Fig. 4:**
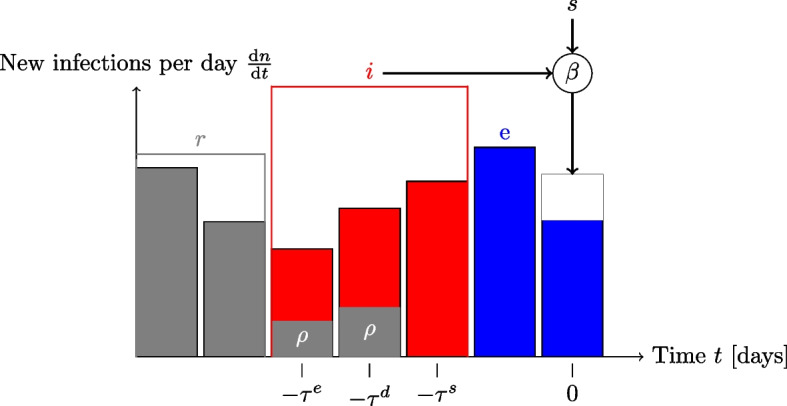
Diagram illustrating the relation between the number of new infections per day and the SEIRS compartments with detected and isolated cases

As detected cases were isolated to avoid transmission, we subtract detected, but yet infectious cases from the effectively infectious fraction, which is easily possible in our model, such that the full model is given by

12 $$\begin{aligned} \frac{\textrm{d}n}{\textrm{d}t}= & \beta (t)\,s(t)\,i(t), \end{aligned}$$

13 $$\begin{aligned} \frac{\textrm{d}p}{\textrm{d}t}= & \beta (t)\,s(t)\,i(t)-\alpha \,p(t), \end{aligned}$$

14 $$\begin{aligned} \frac{\textrm{d}d}{\textrm{d}t}= & \rho (t)\,\frac{\textrm{d}n}{\textrm{d}t}(t-\tau ^d), \end{aligned}$$

15 $$\begin{aligned} s(t)= & 1-p(t), \end{aligned}$$

16 $$\begin{aligned} i(t)= & n(t-\tau ^s)-n(t-\tau ^e)-\left( d_j(t)-d_j(t+\tau ^d-\tau ^e)\right) , \end{aligned}$$

17 $$\begin{aligned} c(t)= & \mu \left( n(t-\tau ^{is}) - n(t-\tau ^{ie})\right) . \end{aligned}$$

Figure 4 illustrates how detected cases reduce the effective infectious compartment and by this also the number of new infections.

#### Parametrization of time-dependent parameters

The transmission rate $\beta (t)$ is the primary factor influencing transmission dynamics over time for any compartmental model. We parameterize $\beta (t)$ to account for the effects of seasonality and behavior, assuming that the transmission rate is the product of a seasonal factor, a behavioral factor and a constant. The behavioral factor is driven by protective behavior enforced by NPIs and self-protective behavior. Thus, we express the transmission rate as:

$$\begin{aligned} \beta (t)=\beta _0\beta _{\text {season}}(t)\, \beta _{\text {behavior}}(t). \end{aligned}$$

**Fig. 5:**
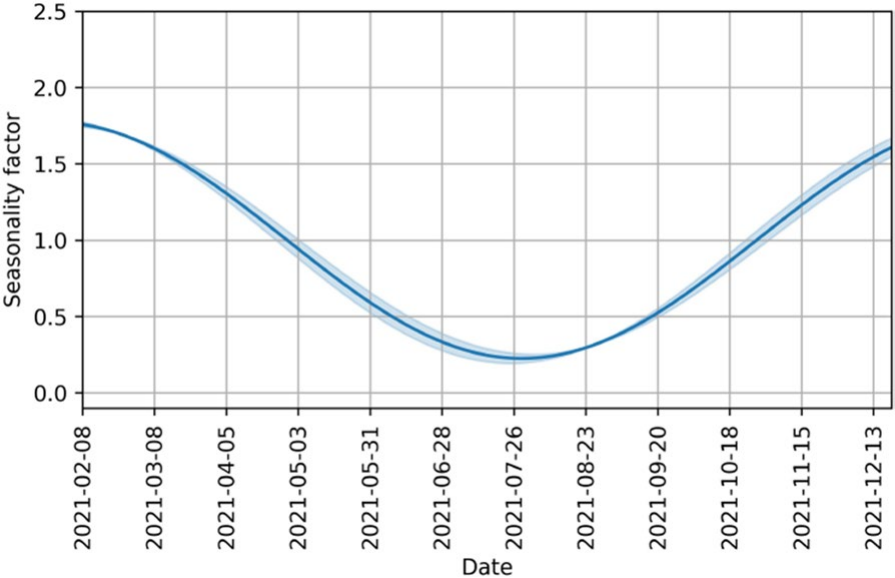
Example of a seasonality factor for the transmission model. It is taken from the seasonality factor identified for Rhineland-Palatinate. The shaded area represents the 95 % confidence interval

For the seasonal factor, we assume a shifted sinusoidal relationship between the date and transmission probability, with a period of one year:

$$\begin{aligned} \beta _{\text {season}}(t)=1+\psi \, \sin \left( 2\pi \frac{t}{365.25}+\phi \right) , \end{aligned}$$

where $\psi$ is the amplitude and $\phi$ is the phase shift of the seasonal variation. This is a classical assumption for seasonally forced infectious diseases [64, 65], also used in several other COVID-19 models [52, 66–69]. Figure 5 illustrates this seasonal factor over time according to our model. The seasonal parameters are identified together with all others as described in the Parameter identification section.

The behavioral factor is split into a factor caused by mandated NPIs and a self-protection factor:

$$\begin{aligned} \beta _{\text {behavior}}(t)=\beta _{\text {NPI}}(t) \,\beta _{\text {self}}(t). \end{aligned}$$

**Fig. 6:**
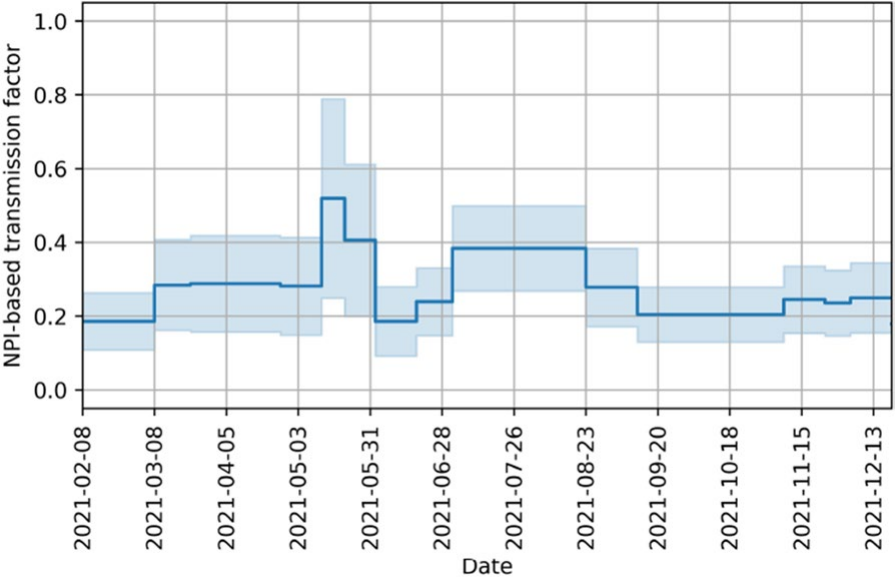
Example of an NPI-based transmission factor. It is taken from the NPI-based transmission factor identified for Rhineland-Palatinate. It illustrates the assumption of a piecewise constant function with jumps where NPIs change. The shaded area represents the 95 % confidence interval

We assume that, in the absence of self-protective behavior, the factor $\beta _{NPI}(t)$ stays the same as long as NPIs do not change. This results in a piecewise constant $\beta _{NPI}(t)$ with jumps at times where NPIs are changed. An example of how the NPI-based transmission factor might look like is shown in Fig. 6. The values of the piecewise constant function are identified alongside the other parameters, while the dates have been set according to the regulations of the states.

For self-protective behavior, we assume that it is influenced by reported ICU occupancy based on the findings by Schulze et al. [2] and Dönges et al. [30]. The second study also shows that self-protective behavior reaches a lower plateau, i.e. even if the ICU occupancy keeps increasing, the contact reduction by self-protection remains constant. In contrast to [30], who use a softplus function to smoothen the relation, we model the transition from no reduction at harmless ICU occupancy levels to maximal reduction by a smooth sigmoidal function:

18 $$\begin{aligned} \beta _{\text {self}}(t)=1-\frac{\eta }{1+\exp \left( -\delta \,(r(t)-\zeta )\right) }. \end{aligned}$$

Here, $\eta$ represents the maximum possible contact reduction, $\delta$ is the feedback rate, *r*(*t*) is the reported ICU occupancy at time *t*, and $\zeta$ is the feedback center.

**Fig. 7:**
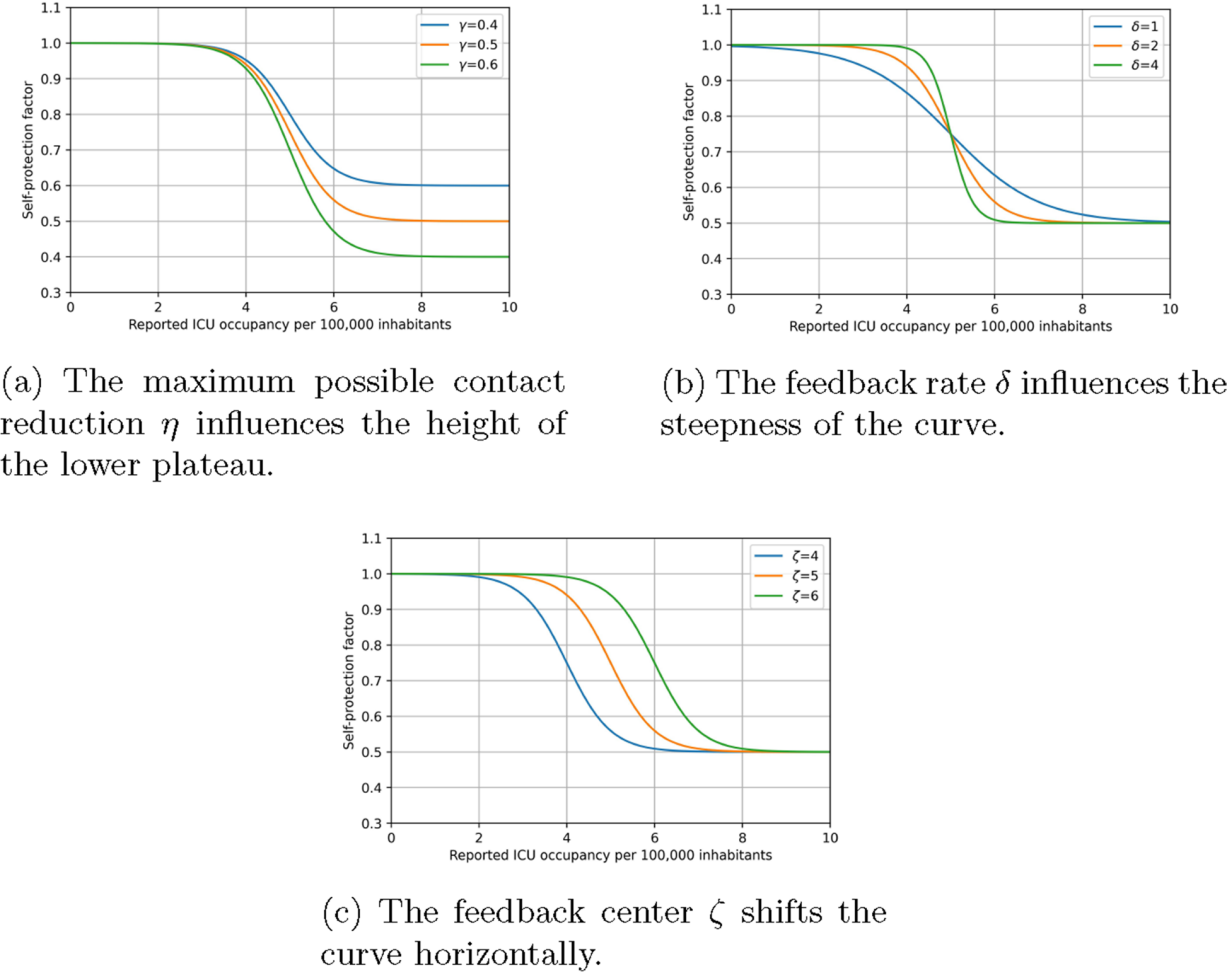
The self-protection factor can be adapted by three parameters, the maximum possible contact reduction $\eta$, the feedback rate $\delta$ and the feedback center $\zeta$. The orange line corresponds to \eta=0.5, \delta=2, \zeta=5. The blue lines correspond to a shifted parameter of \eta=0.4, \delta=1, \zeta=4, respectively. The green lines correspond to a shifted parameter of \eta=0.6, \delta=4, \zeta=6, respectively.

The feedback rate affects the steepness of the sigmoidal curve, while the feedback center indicates the reported incidence at which half of the maximum contact reduction occurs. Figure 7 illustrates the relationship outlined in Eq. (18) for various parameter configurations.

Figure 14 in the Results section shows the identified values over time and illustrates how the total transmission rate is influenced by the contributing factors.

As jointly estimating $\beta _0$ and the factors is not possible, we have set $\beta _0=2.788$. This estimate is the product of the pre-pandemic average number of contacts per day among all ages in Germany retrieved from the SOCRATES tool [70] based on data from a study by Mossong et al. [71] and the estimated secondary attack rate of SARS-CoV-2 [72].

Additionally, the detection rate $\rho (t)$ is also parameterized as piecewise constant. We assume that changes are aligned with the beginning and end of school vacations, as previous findings indicated that school testing (and its absence during vacations) significantly influenced the detection rate in these German states [47].

#### Full model with cross-immunity between variants

**Fig. 8:**
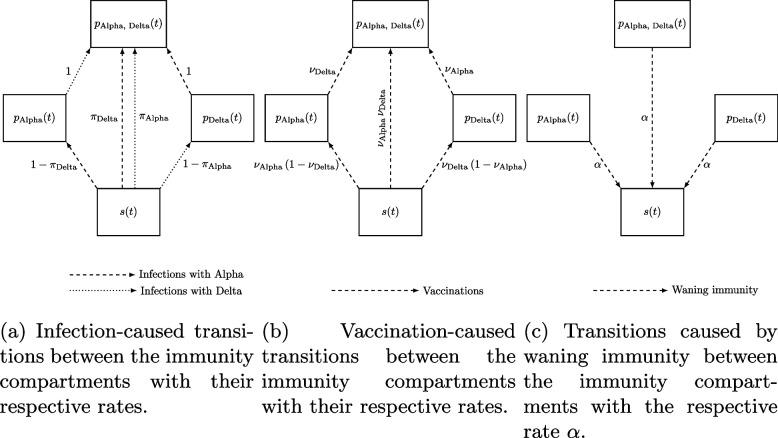
Flowchart representing the transitions between immunity compartments caused by infections, vaccinations and waning immunity with their respective rates. The rates $\pi _{\text {Alpha}}$ and $\pi _{\text {Delta}}$ are the respective cross-immunity factors between the two variants. The vaccine effectiveness against variant *j* is denoted by $\nu _j$. The parameter $\alpha$ is called rate of waning immunity. Dashed arrows correspond to infections with the Alpha variant, or vaccinations, or waning immunity, respectively. Dotted arrows correspond to infections with the Delta variant

As there were two predominant variants of SARS-CoV-2 during the study period, we use the extension of the model to two variants. For this, the immunity compartment *p*(*t*) is split into compartments with immunity against the Alpha variant $p_{\text {Alpha}}(t)$, immunity against the Delta variant $p_{\text {Delta}}(t)$ and immunity against both variants $p_{\text {Alpha, Delta}}(t)$. For each infection with the Alpha variant, there is a probability $\pi _{\text {Delta}}$ that immunity against the Delta variant is also obtained and respectively for infections with the Delta variant. In the same way, there is a vaccine effectiveness $\nu _{\text {Alpha}}$ against the Alpha variant and one, $\nu _{\text {Delta}}$, against the Delta variant. A flowchart of the protection compartments and the transitions between them can be seen in Fig. 8.

Additionally, the Delta variant has a relative transmissibility factor $\omega _{\text {Delta}}$, which is multiplied by the transmission rate, while for Alpha, it is set to $\omega _{\text {Alpha}}=1$. It is important to note that the basic SEIRS-like transmission model is not changed, but additionally we account for different immunity states and count infections with each variant separately. A full derivation of the two-variant model and its mathematical analysis can be found in [58]. The equations obtained for the model are

$$\begin{aligned} \frac{\textrm{d}n_j(t)}{\textrm{d}t}= & \beta (t)\,\left[ \omega _j\,s_j(t)\,i_j(t)\right] ,\quad \, j\in \{\text {Alpha, Delta}\}, \\ \frac{\textrm{d}d_j(t)}{\textrm{d}t}= & \rho (t)\,\dot{n}_j(t-\tau ^d),\quad \quad \quad j\in \{\text {Alpha, Delta}\},\\ \frac{\textrm{d}p_{\text {Alpha}}(t)}{\textrm{d}t}= & -\alpha \,p_{\text {Alpha}}(t) \\ & +(1-\pi _{\text {Delta}})\left[ \beta (t)\,s(t)\,i_{\text {Alpha}}(t)\right] -\beta (t)\,\omega _{\text {Delta}}\,p_{\text {Alpha}}(t)\,i_{\text {Delta}}(t)\\ & +\nu _{\text {Alpha}}\,(1-\nu _{\text {Delta}})\,s(t)\,v(t-\tau ^p)-\nu _{\text {Delta}}\,p_{\text {Alpha}}(t)\,v(t-\tau ^p),\\ \frac{\textrm{d}p_{\text {Delta}}(t)}{\textrm{d}t}= & -\alpha \,p_{\text {Delta}}(t) \\ & +(1-\pi _{\text {Alpha}})\left[ \beta (t)\,\omega _{\text {Delta}}\,s(t)\,i_{\text {Delta}}(t)\right] -\beta (t)\,p_{\text {Delta}}(t)\,i_{\text {Alpha}}(t)\\ & +\nu _{\text {Delta}}\,(1-\nu _{\text {Alpha}})\,s(t)\,v(t-\tau ^p)-\nu _{\text {Alpha}}\,p_{\text {Delta}}(t)\,v(t-\tau ^p),\\ \frac{\textrm{d}p_{\text {Alpha, Delta}}(t)}{\textrm{d}t}= & -\alpha \, p_{\text {Alpha, Delta}}(t) \\ & +\pi _{\text {Delta}}\,\left[ \beta (t)\, s(t)\,i_{\text {Alpha}}(t)\right] +\beta (t)\,p_{\text {Delta}}(t)\,i_{\text {Alpha}}(t)\\ & +\pi _{\text {Alpha}}\,\left[ \beta (t)\,\omega _{\text {Delta}}\,s(t)\,i_{\text {Delta}}(t)\right] +\beta (t)\,\omega _{\text {Delta}}\,p_{\text {Alpha}}(t)\,i_{\text {Delta}}(t)\\ & +\nu _{\text {Alpha}}\,\nu _{\text {Delta}}\,s(t)\,v(t-\tau ^p)+\nu _{\text {Delta}}\,p_{\text {Alpha}}(t)\,v(t-\tau ^p)\\ & +\nu _{\text {Alpha}}\,p_{\text {Delta}}(t)\,v(t-\tau ^p), \end{aligned}$$

with the following quantities given by algebraic expressions:

$$\begin{aligned} \begin{array}{ll} i_j(t)=n_j(t-\tau ^s)-n_j(t-\tau ^e)-\left[ d_j(t)-d_j(t+\tau ^d-\tau ^e)\right] ,& j\in \{\text {Alpha, Delta}\},\\ c_j(t)= \mu _j \,\left( n_j(t-\tau ^{is}) - n_j(t-\tau ^{ie})\right) ,& j\in \{\text {Alpha, Delta}\},\\ s(t)=1-p_{\text {Alpha}}(t)-p_{\text {Delta}}(t)-p_{\text {Alpha, Delta}}(t),& \\ s_j(t)=1-p_{\text {Alpha, Delta}}(t)-p_{j}(t),& j\in \{\text {Alpha, Delta}\}.\\ \end{array} \end{aligned}$$

Table 1 shows all model compartments and parameters with their symbol and interpretation.

**Table 1:** All model compartments and parameters with their symbol and interpretation

Symbol	Interpretation
$n_j(t)$	Cumulated number of infections with variant *j* at time *t*
$d_j(t)$	Cumulated number of detected infections with variant *j* at time *t*
$p_j(t)$	Fraction of population immune solely against variant *j* at time *t*
$p_{\text {Alpha, Delta}}(t)$	Fraction of population immune against both variants at time *t*
$i_j(t)$	Fraction of population infectious with variant *j* at time *t*
$c_j(t)$	Fraction of population in ICU with variant *j* at time *t*
*s*(*t*)	Fraction of population susceptible to all variants at time *t*
$s_j(t)$	Fraction of population susceptible to variant *j* at time *t*
$\beta (t)$	Transmission rate at time *t*
$\rho (t)$	Detection rate at time *t*
*v*(*t*)	Vaccination rate at time *t*
$\omega _{\text {Delta}}$	Relative transmissibility of Delta compared to Alpha
$\alpha$	Rate of waning immunity
$\pi _j$	Probability of acquiring cross-immunity against variant *j*, when infected with the other variant
$\nu _j$	Vaccine effectiveness against variant *j*
$\mu _j$	Fraction of cases with variant *j* going to ICU
$\tau ^d$	Delay from infection to detection
$\tau ^p$	Delay from vaccination to acquiring immunity
$\tau ^s$	Delay from infection to start of infectiousness
$\tau ^e$	Delay from infection to end of infectiousness
$\tau ^{is}$	Delay from infection to ICU admission
$\tau ^{ie}$	Delay from infection to ICU release

### Parameter identification

Determining the impact of self-protection on the transmission dynamics of SARS-CoV-2 presents a significant challenge. This complexity arises from the fact that both, the NPI-based transmission reduction and the self-protection, are factors of a single effective parameter in our model, specifically the transmission rate $\beta (t)$, and we lack additional reliable and objective data to differentiate between them.

It is important to recognize that in our parameterization, the values of the NPI-based transmission factor influence the transmission rate for a limited period of time, while those related to self-protection are the same for all times. This structural difference allows us to identify parameters for both effects in our model. In the following, we outline the approach for identifying the parameters within our model.

Our goal is to identify parameters that ensure our model aligns optimally with the data described in the Data description and sources section. To differentiate between discrete real-world data and continuous model variables, we denote data by upper case letters and an index for the time instead of lower case letters with an argument for model variables. Let $D_t$ represent the new cases reported for day *t*, and $C_t$ the number of reported ICU patients with COVID-19 on day *t*. Let *q* denote the vector of parameters to be identified and $d(t;\,q),\,c(t;\,q)$ the model quantities $d(t),\,c(t)$ which depend on the parameter *q*. The squared sum of model errors can be expressed as:

19 $$\begin{aligned} f(q)=\sum \limits _t \left( \frac{\left[ d(t;\,q)-d(t-1;\,q)\right] - D_t}{\sigma _d} \right) ^2 + \left( \frac{c(t;\,q) - C_t}{\sigma _c} \right) ^2, \end{aligned}$$

where $\sigma _d$ and $\sigma _c$ are the standard deviations of the measurement errors of detected cases and ICU patients, respectively. Note that only the fraction $\chi = \frac{\sigma _d^2}{\sigma _c^2}$ modifies the optimization result. We chose $\chi =10$ to cope for the different magnitudes of the measured quantities. Minimizing this function is a weighted nonlinear least squares method [73].

To minimize the function *f*(*q*), we employ a Gauss-Newton algorithm [74]. The gradient of each quantity with respect to the parameters being identified is calculated using automatic differentiation [75]. By using automatic differentiation, we also find the sensitivity matrix *S*, which relates measurement errors $\varepsilon _{(d,c)}$ to parameter modifications $\varepsilon _q$ as $\varepsilon _q = S \, \varepsilon _{(d,c)}$. Assuming independent normally distributed measurement errors with zero mean and diagonal covariance matrix $C_{(d,c)} = \text{ diag } \left( \sigma _i^2 \right) = \text{ diag }\left( \sigma _d^2,\dots ,\sigma _d^2,\sigma _c^2,\dots ,\sigma _c^2\right)$, we can compute the covariance matrix of the estimation error as

20 $$\begin{aligned} C_q = S \, C_{(d,c)} \, S^t \; \text{ and } \; \sigma (q_i) = \sqrt{{C_q}_{ii}} \; . \end{aligned}$$

These standard deviations are used for the confidence intervals of the parameter estimates. The underlying standard deviations of the measurement errors are determined as empirical standard deviations of the real measurements from a curve fit.

Identifying the parameters for the self-protection factor requires initial values that are in a neighborhood of the optimal solution as in general only the product of $\beta _{\text {NPI}}$ and $\beta _{\text {self}}$ is well identifiable. Therefore, we determine suitable starting values for the self-protection parameters using a grid-search method. Through this combination of techniques and the distinction between parametrization for $\beta _{\text {NPI}}$ local in time and parameterization for $\beta _{\text {self}}$ global in time, we successfully identify parameters for both the NPI-based transmission factor and the self-protection factor, as well as other model parameters.

Nevertheless, there are some parameters that cannot be identified solely based on case and ICU data. The vaccination rate *v*(*t*) is set based on the data available [42]. We computed the initial values for the immunity compartments by data from a German seroprevalence study [76] and data on the effectiveness of a previous infection against a new infection with the Alpha or Delta variant [77]. If *p* is the total fraction of the population with antibodies as given by [76] ($p=0.085$) and $\pi _{\text {Alpha}}$ and $\pi _{\text {Delta}}$ denote the effectiveness of a previous infection against reinfection with the indexed variant with data from [77] ($\pi _{\text {Alpha}}=0.895$, $\pi _{\text {Delta}}=0.900$), we set

21 $$\begin{aligned} p_{\text {Alpha}}(0)= & p\,\pi _{\text {Alpha}}\,(1-\pi _{\text {Delta}}), \end{aligned}$$

22 $$\begin{aligned} p_{\text {Delta}}(0)= & p\,(1-\pi _{\text {Alpha}})\,\pi _{\text {Delta}}, \end{aligned}$$

23 $$\begin{aligned} p_{\text {Alpha, Delta}}(0)= & p\,\pi _{\text {Alpha}}\,\pi _{\text {Delta}}. \end{aligned}$$

**Fig. 9:**
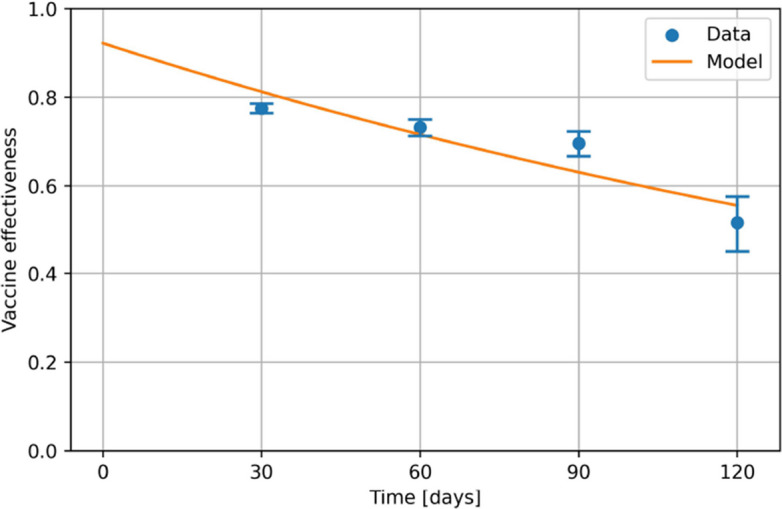
Interpolation of the vaccine effectiveness over time. The exponential parameter $\alpha$ of the model function $\nu (t)=\nu _{0}\exp (-\alpha t)$ is used in the transmission model

For the waning rate $\alpha$, we performed an exponential fit of the data in [78], i.e. finding $\nu _0$ and $\alpha$ of the function

24 $$\begin{aligned} \nu (t)=\nu _{0}\exp (-\alpha t) \end{aligned}$$

with the best approximation of the data using a nonlinear least-squares method. The resulting interpolation can be seen in Fig. 9.

A table of all parameters that were not identified but set using literature values can be found in Table 2.

**Table 2:** Parameters and initial values which have been set according to literature values

Initial value/parameter	Value	Source(s)
$p_{\text {Alpha}}(0)$	0.0076	[76, 77], (21)
$p_{\text {Delta}}(0)$	0.0080	[76, 77], (21)
$p_{\text {Alpha, Delta}}(0)$	0.0685	[76, 77], (21)
$\alpha$	0.0041	[78]
$\pi _{\text {Alpha}}$	0.895	[77]
$\pi _{\text {Delta}}$	0.900	[77]
$\nu _{\text {Alpha}}$	0.937	[63]
$\nu _{\text {Delta}}$	0.880	[63]
$\tau ^d$	7.1	[79]
$\tau ^p$	14	[62]
$\tau ^s$	4.7	[79]
$\tau ^e-\tau ^s$	5	[60]
$\tau ^{ie}-\tau ^{is}$	13.5	[80]

## Results

In this section, we present the results of our analysis. First, we assess the fit quality of our model across the four German states included in the study. Then, we report the optimal parameter values found, for both viral and behavioral parameters. Afterwards, we present counterfactual simulations that consider only the effect of NPIs or self-protection, neglecting the other one. Values in parentheses throughout this section denote the standard errors of the corresponding quantities and the shaded areas in the figures represent the 95 % confidence intervals of parameters.

### Model fitting

**Fig. 10:**
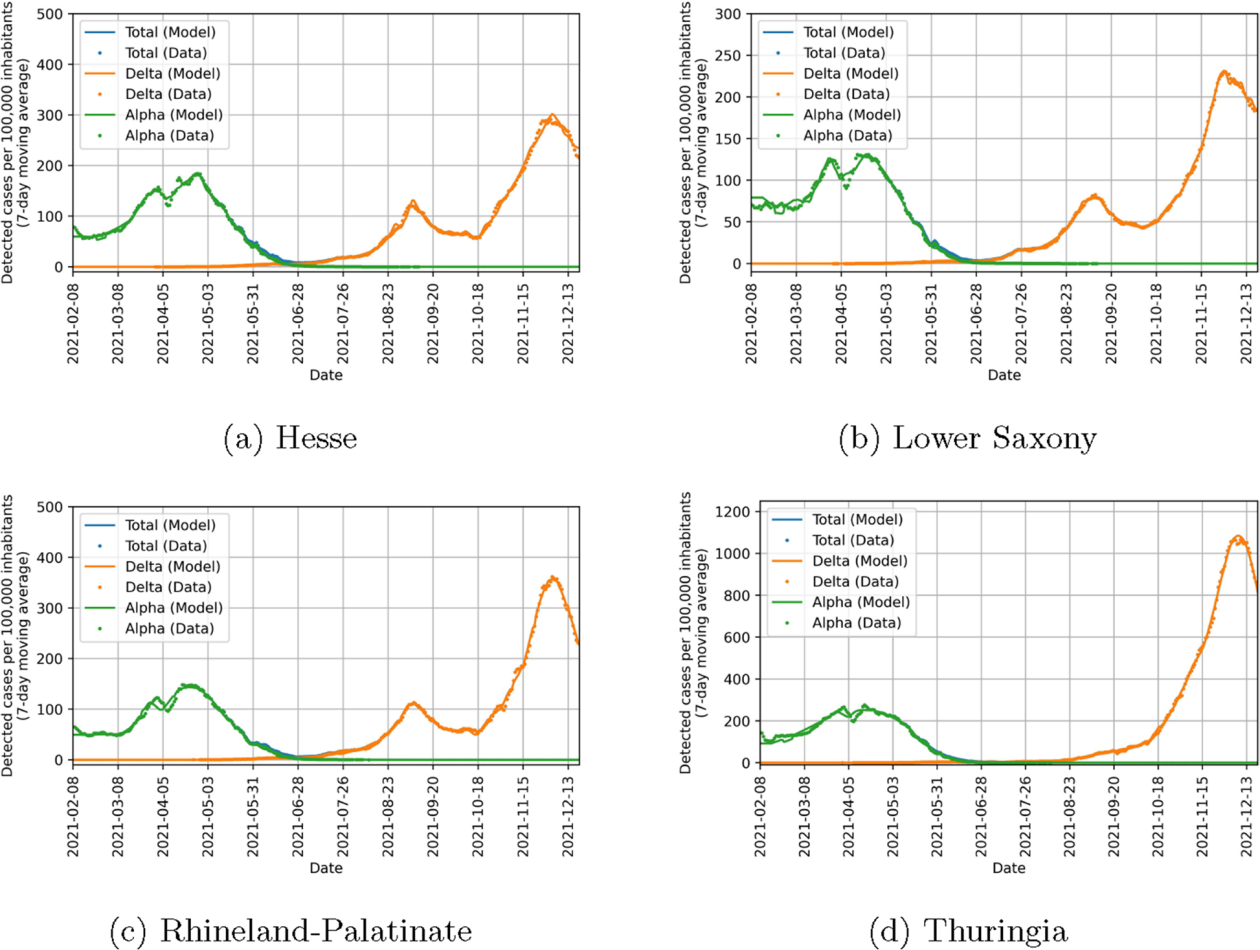
Comparison of data and model output for detected COVID-19 cases per 100,000 inhabitants (7-day moving average) in different states of Germany. Lines correspond to model quantities and points to reported data. Alpha cases are represented in green, Delta cases in orange and cumulated cases in blue

**Fig. 11:**
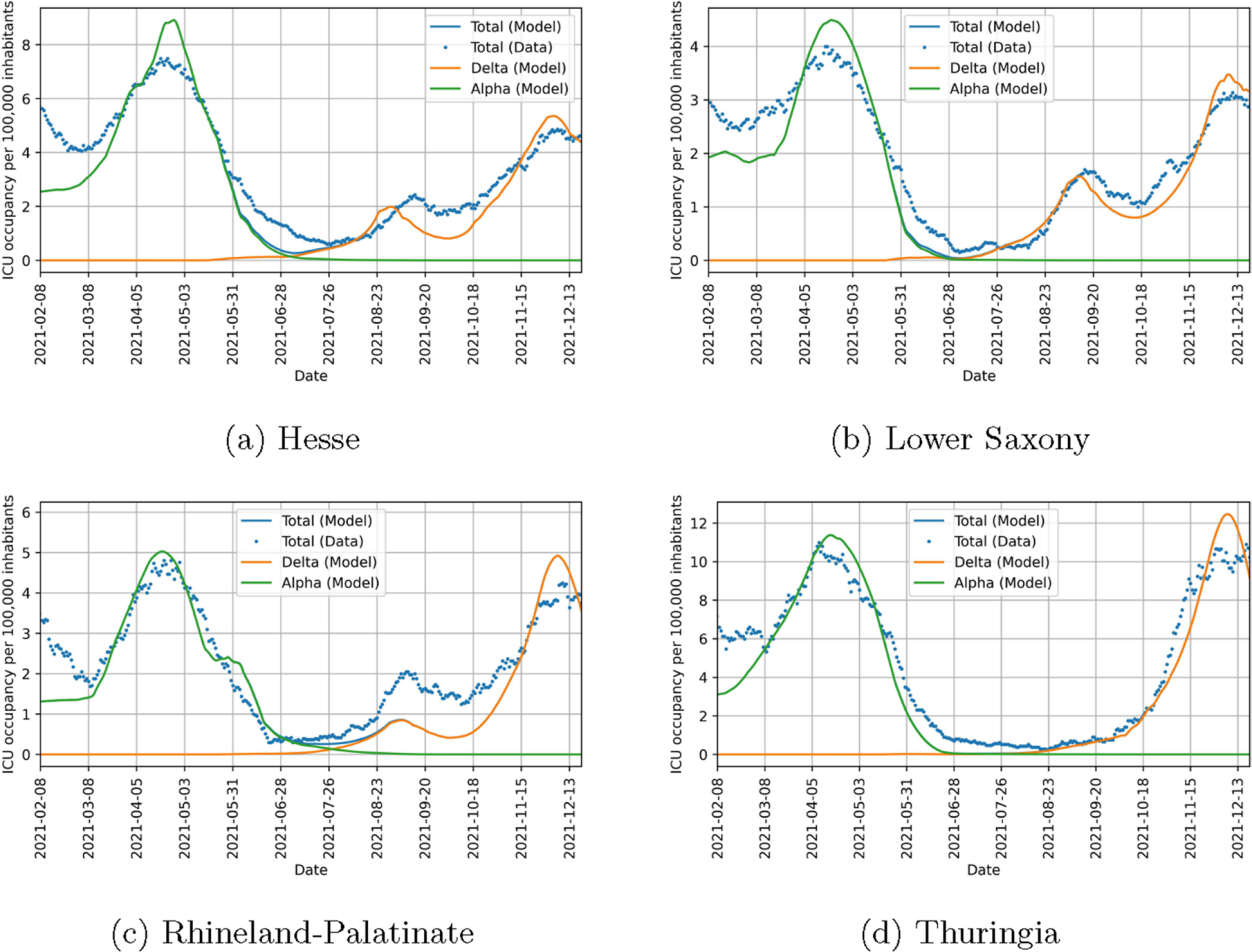
Comparison of data and model output for ICU beds occupied by COVID-19 patients per 100,000 inhabitants in different states of Germany. Lines correspond to model quantities and points to reported data. Alpha patients are represented in green, Delta patients in orange and cumulated patients in blue

**Fig. 12:**
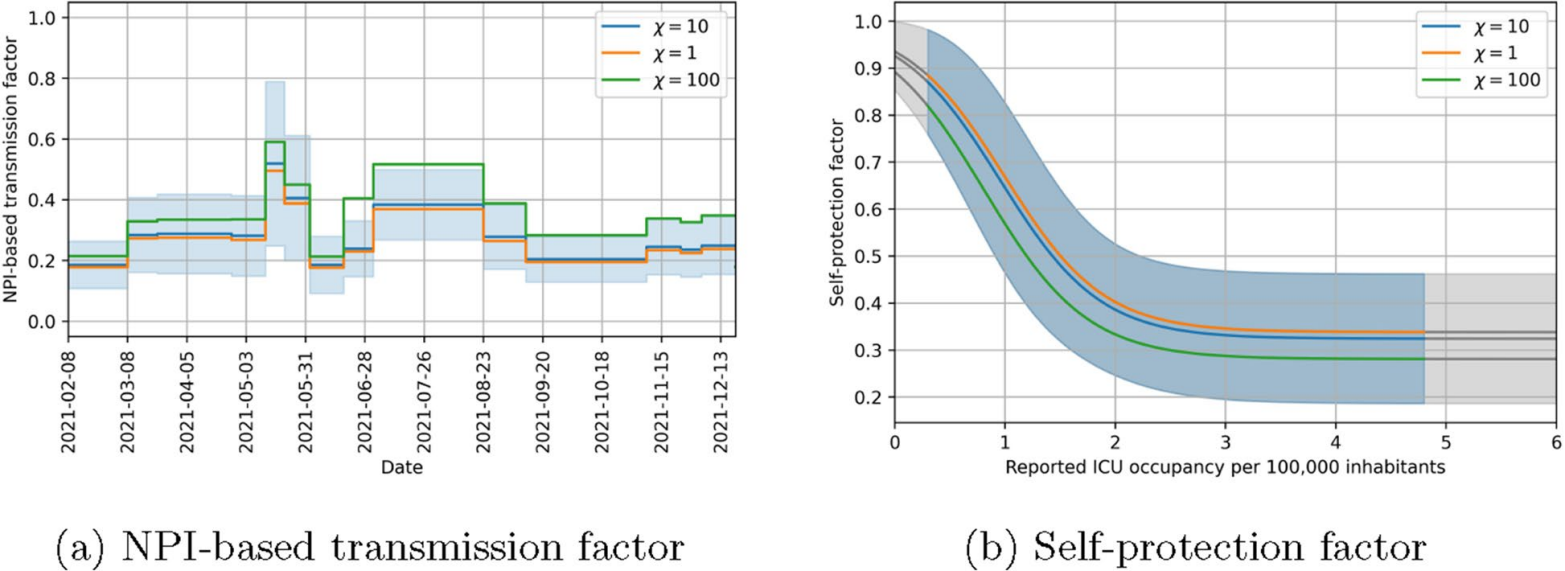
NPI-based transmission factor and self-protection factor for different values of $\chi$ in Rhineland-Palatinate. Blue curves correspond to the default value of \chi=10, orange ones to \chi=1 and green ones to \chi=100

Before considering the implications of the parameters found, we look at how good our model can fit the data in the four states included in the study. Figure 10 shows that there is a strong agreement between the observed data and the model output in detected cases. There is also a good agreement in ICU beds occupied between data and model as seen in Fig. 11. However, this agreement is not as good as for detected cases. We could improve the fit quality of ICU beds occupied by changing the value of $\chi$ in Eq. (19) while simultaneously decreasing the fit quality of detected cases. Finding an optimal balance between these was not in the focus of our study and the chosen value of $\chi$ yields satisfactory results, so we chose not to optimize it. Figure 12 shows that the behavioral parameters for a wide range of $\chi$ are within the uncertainty intervals of these parameters for the chosen $\chi =10$. As this uncertainty is already recognized in the counterfactual simulations, we conclude that they are also valid for other values of $\chi$. Furthermore, we expect a difference between our model and the observed ICU data at the beginning of the study period. This is due to people which have been infected in the pre-Alpha wave and are still in ICU. Overall, our model yields satisfactory fit results showing that it can represent well the infection dynamics during the study period.

### Virus parameters

In this section, we present the values of the model parameters related to the virus characteristics found by parameter identificaton as described in the Parameter identification section. We identified the parameters independently for each state to enable comparison and cross-validation. Table 3 presents all these values including their uncertainty. We observe that the values for the increased transmissibility of the Delta variant compared to the Alpha variant, $\omega _{\text {Delta}}$, is really close among all states. The ratio of infections going to ICU differs between states, but is at least of the same order of magnitude. Furthermore, we note that this ratio is smaller for Delta infections than for Alpha infections. The values for the delay between infection and ICU admission vary across states and variants and cannot be determined with the same precision. The results for the parameters of the seasonality are close to each other for the different states with its minimum in July/August and the peak in January/February, correspondingly. Only in Thuringia, the curve is shifted having its minimum in early September and the maximum in March, but with a similar amplitude. Figure 5 shows the identified seasonality factor in Rhineland-Palatinate.

**Table 3:** Optimal values and their uncertainty for virus parameters found by parameter identification

Parameter	Hesse	Lower Saxony	Rhineland-Palatinate	Thuringia	Scaling factor
$\omega _{\text {Delta}}$	2.01 (0.03)	1.91 (0.03)	2.05 (0.06)	1.88 (0.13)	1
$\mu _{\text {Alpha}}$	1.18 (0.02)	0.70 (0.02)	0.71 (0.03)	0.93 (0.04)	1e-2
$\mu _{\text {Delta}}$	5.13 (0.23)	7.50 (0.27)	2.52 (0.12)	6.25 (0.21)	1e-3
$\tau ^{is}_{\text {Alpha}}$	9.49 (0.59)	9.70 (0.79)	2.19 (1.34)	4.53 (1.32)	1
$\tau ^{is}_{\text {Delta}}$	4.58 (1.00)	10.49 (1.22)	6.57 (1.29)	3.84 (1.01)	1
$\psi$	8.58 (0.09)	5.74 (0.19)	7.74 (0.15)	6.92 (0.50)	1e-1
$\phi$	2.31 (0.02)	2.23 (0.03)	2.26 (0.01)	1.64 (0.05)	1

Reference day with t=0 is March 8, 2020 for the seasonality factor

### Behavioral parameters

**Fig. 13:**
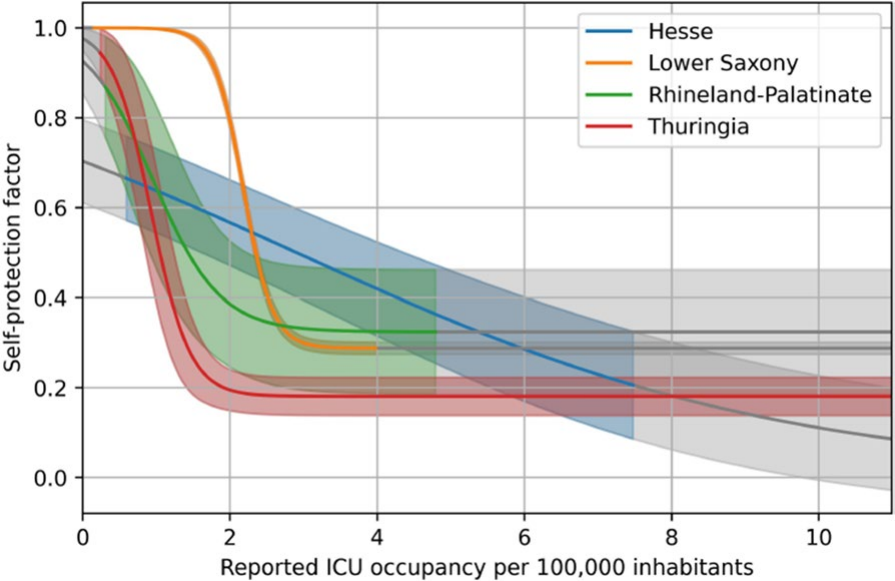
Optimal curve for the self-protection factor $\beta _{\text {self}}$ in different states of Germany. Low values of the self-protection factor indicate high levels of self-protection. Grey areas mark extensions of the curves in regions where no ICU occupancy was reported during the simulation period. The blue line corresponds to the curve for Hesse, the orange one for Lower Saxony, the green one for Rhineland-Palatinate and the red one for Thuringia

In this section, we focus on the behavioral parameters identified in the different states, especially those related to the self-protection factor $\beta _{\text {self}}$ as described in Eq. (18). The identified parameter values of Eq. (18) can be found in Table 4 as well as the range of ICU occupancy per 100,000 inhabitants during the timeframe considered in the four states. Note that these parameters cannot be interpreted biologically. They are defined indirectly as shape parameters of the self-protection curve $\beta _{\text {self}}$, which contributes to the effective parameter $\beta (t)$. Consequently, the parameter values differ across the states, though they remain of the same order of magnitude. Moreover, the range of reported ICU occupancy varies significantly between the states, with substantially higher ranges in Hesse and Thuringia than in Lower Saxony and Rhineland-Palatinate. This can also be seen in the data in Fig. 11, where different scales of the y-axis were used in the different states. Figure 13 shows the identified feedback curves in the different states and their confidence interval. We observe that the curves for Rhineland-Palatinate and Thuringia are quite similar, while the curve for Lower Saxony is displaced to higher ICU occupancies. The curve for Hesse is flatter than the others, but its confidence interval would also allow for steeper curves. A common feature of all curves is that the maximal reduction obtained with the ICU occupancies observed is between 67.6 (6.9) % in Rhineland-Palatinate and 81.9 (2.1) % in Thuringia, which is in the same order of magnitude.

**Fig. 14:**
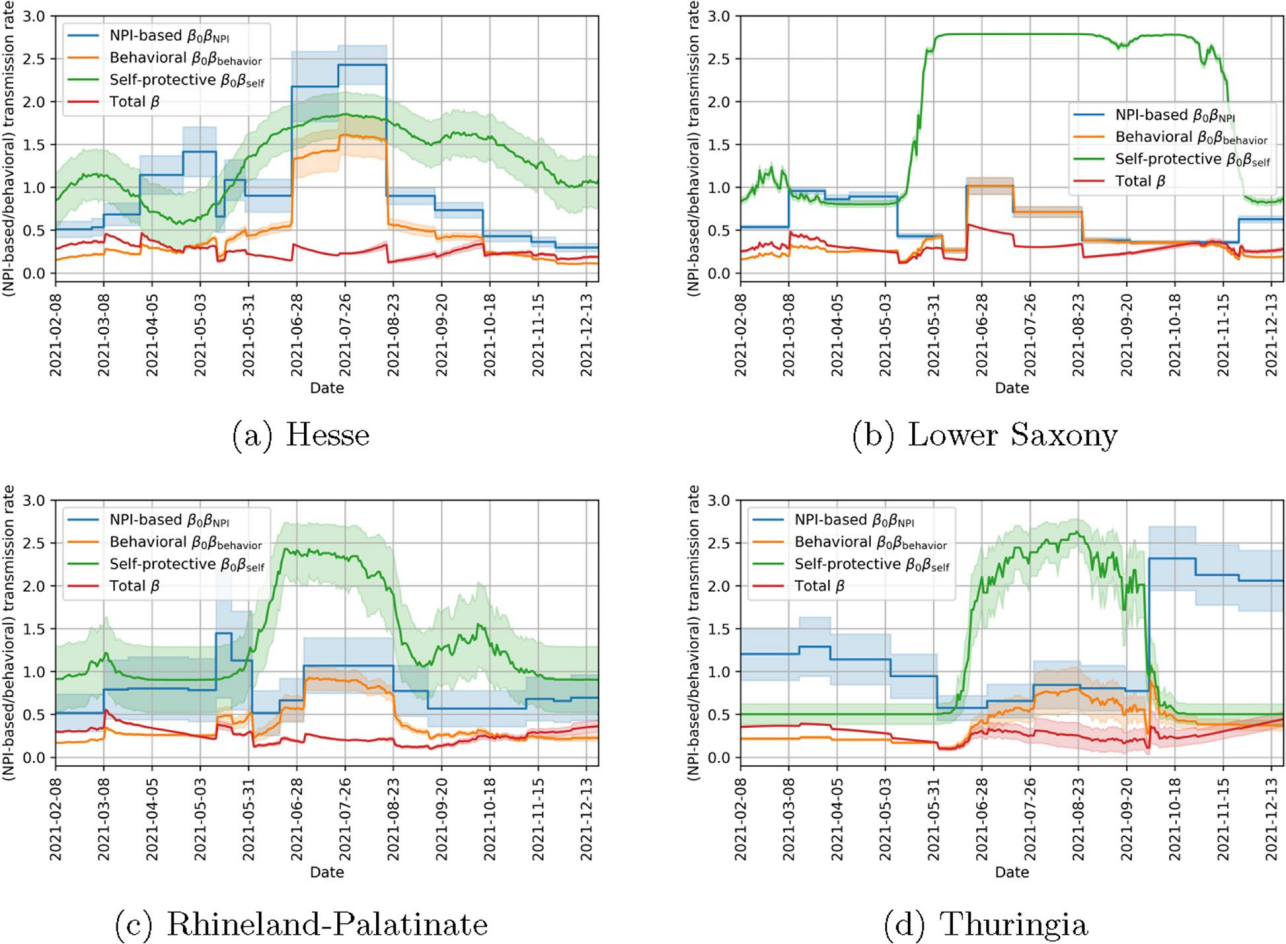
NPI-based, behavioral and total (including seasonality) transmission rate and its standard errors in different states of Germany. The blue curves correspond to the NPI-based transmission rate, the orange ones to the behavioral transmission rate, the green one to the self-protective transmission rate and the red one to the overall transmission rate including seasonality

Figure 14 shows the identified transmission rate as well as the contributing factors. We observe that the behavioral transmission rate is highest during the summer months in all states. This does not lead to the highest overall transmission rate, however, due to the seasonality of the virus. Another observation is the increasing uncertainty of the factors of the transmission rate. While the overall transmission rate can be determined quite accurately, the NPI-based transmission rate $\beta _0\beta _{\text {NPI}}$ and the self-protective one $\beta _0\beta _{\text {self}}$ have a much higher uncertainty and the behavioral transmission factor as an intermediate factor has an intermediate uncertainty.

**Table 4:** Optimal values and their uncertainty for behavioral parameters and range of reported ICU occupancy

Parameter	Hesse	Lower Saxony	Rhineland-Palatinate	Thuringia	Scaling factor
$\eta$	9.95 (0.53)	7.12 (0.07)	6.76 (0.69)	8.19 (0.21)	1e-1
$\delta$	0.30 (0.04)	4.99 (0.21)	2.20 (0.24)	3.78 (0.53)	1
$\zeta$	2.87 (0.56)	2.18 (0.01)	0.95 (0.22)	0.93 (0.10)	1
*r*(*t*)	0.59–7.48	0.15–4.00	0.29–4.81	0.24–10.99	1

### Counterfactual simulations

To illustrate the impact of self-protection on transmission dynamics, we have conducted two counterfactual simulations per state, one without self-protection and one without NPIs. For the simulations without self-protection, we set the self-protection factor $\beta _{\text {self}} = 1$. For those without NPIs, we set the NPI-based transmission factor $\beta _{\text {NPI}}=1$ and determined $\beta _{\text {self}}$ based on the ICU occupancy computed by the model itself. All other parameters were left unchanged and their uncertainties were used to evaluate the uncertainty of the counterfactuals.

**Fig. 15:**
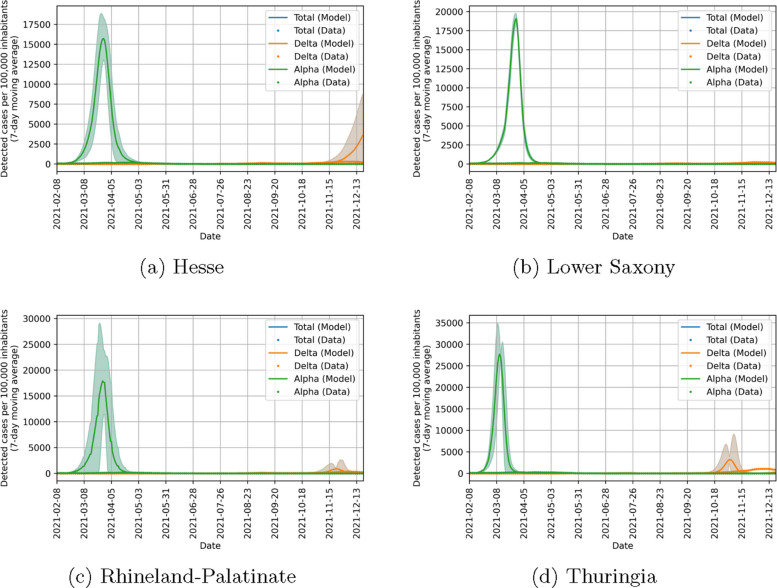
Counterfactual simulations (without self-protection) for detected COVID-19 cases per 100,000 inhabitants (7-day moving average) in different states of Germany. Note the different scales on the y-axis. Lines correspond to model quantities and points to reported data. Alpha cases are represented in green, Delta cases in orange and cumulated cases in blue

**Fig. 16:**
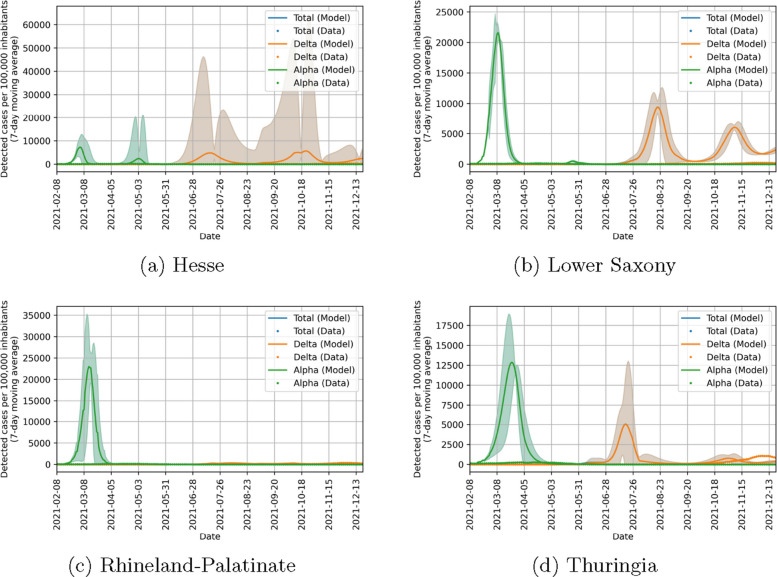
Counterfactual simulations (without NPIs) for detected COVID-19 cases per 100,000 inhabitants (7-day moving average) in different states of Germany. Note the different scales on the y-axis. Lines correspond to model quantities and points to reported data. Alpha patients are represented in green, Delta patients in orange and cumulated patients in blue

Figure 15 shows the counterfactual simulations without self-protection. We see that, while the exact trajectory is uncertain, the number of infections in the counterfactual simulations are always orders of magnitude larger than those observed, especially for the Alpha wave. The Delta wave is limited compared to the Alpha wave due to the immunization of large portions of the population during the Alpha wave. Despite this, it still exceeds the real observed values. Note that this scenario is purely academic, as not only people refuse self-protection but also the government does not increase measures in case of high infection numbers.

Figure 16 shows the counterfactual simulations without NPIs. Also in this case, the number of infections in the counterfactual scenario exceed the observed ones by orders of magnitude, while the exact transmission dynamics is quite uncertain. More specifically, we observe that the highest observed reductions due to self-protection were not able to reduce the spreading of the Alpha variant under critical levels. Only for Hesse, this was partially the case, resulting in a second, smaller Alpha wave. As for the counterfactuals without self-protection, the Delta waves are smaller than the Alpha waves due to the immunity in the population.

## Discussion

In this section, we want to discuss the results presented in the Results section. To this end, it is structured in the same way and the findings are discussed accordingly. Finally, a subsection on policy implications of the findings is added.

### Model fitting

We have seen that our model reproduces the data in four different states of Germany very well. This means that our model captures the essential effects of the infectious disease dynamics. However, good fit quality is necessary, but not sufficient for a good and realistic model. Other models were also able to reproduce the German data with other parametrizations and a different focus in their studies [81–84]. The quality of our model does however not only rely on the fit quality, but the parametrizations chosen have been validated in other studies as well (see the Transmission model section for references). Self-protective behavior is the focus of our study and its functional form has been described and validated by survey data in [30]. The validation actually uses survey data from 2021 (and 2020) in Germany, which covers the model period of our study, making it especially applicable to our model.

Despite its validation, our model also neglects some important aspects that influenced infection disease dynamics in Germany. Those include age, gender and regional heterogeneity [85–87], contact networks, individual behavior [88] and large-scale events [89, 90]. We have neglected large-scale events because of their limited impact compared to regular transmission, especially during the high prevalence periods in spring and autumn 2021. Heterogeneity and contact networks are not included in our model, mainly due to identifiability of parameters. Using a homogeneous mixing assumption, which is classical for compartmental models [57, 91], allows us to identify most parameters purely based on infection data. Regional heterogeneity has been incorporated by modeling four states of Germany separately. Individual behavior is not included in our model because we focus on population-wide estimates of the self-protection. While individual behavior can be influenced by multiple factors [2, 92, 93], Dönges et al. [30] showed a clear correlation between ICU occupancy and self-protective behavior on a population level. Using this approach, we were also able to identify the behavioral parameters based on infection data.

Future studies could investigate how such heterogeneities in the population interact with joint behavioral response by mandated NPIs and self-motivated behavioral change.

### Virus parameters

Focusing on a model with relatively few parameters, we were able to identify them. Some of those were related to virus characteristics and can be compared to other studies. The increased transmissibility of the Delta variant compared to the Alpha variant shows that the Delta variant not only became the dominant variant because of partial immune escape, but also an inherent advantage in transmission. Our estimates of the increased transmissibility align with a study conducted in New England [94]. While being identified independently among the four states, the closeness of the parameter among them further supports the evidence that we were able to identify this parameter correctly.

The infection-ICU ratios identified for the four states differ more, but have the same order of magnitude. However, there are also reasons that explain why one cannot expect them to be very close. The risk of ICU admission given a COVID-19 infection depends on several factors, especially age, gender and comorbidities [80, 95, 96], which are not equally distributed among German states. Furthermore, the protective effect of vaccination is only modeled against infection which is lower than against severe infection [97]. Therefore, also differences in vaccination uptake among the states can lead to different estimates of the infection-ICU ratio. This also explains why we find a lower ratio for the Delta variant than for the Alpha variant despite studies showing that the Delta variant was more severe than the Alpha variant [98, 99]. However, during the Delta wave a large portion of the population was already vaccinated which was not the case during the Alpha wave. As our estimate was not corrected for this, the infection-ICU ratio is estimated lower for the Delta variant. This aligns with another study in Spain that compares ICU admission risks during the Alpha and Delta period [100].

Another parameter that was not consistent across states was the delay between infection and ICU admission. This parameter however only represents a time shift of the ICU curve. As the actual reported ICU occupancy is used as an input for the self-protection function $\beta _{\text {self}}$, see Eq. (18), it does not significantly impact our findings regarding the behavioral aspects as the focus of our study.

The seasonality factor, which is influenced by virus characteristics as well as behavioral differences throughout the year, is found to be consistent among the states with a slight shift in Thuringia. Again, this supports our findings. The amplitude is higher than in [52]. This could be attributed to two aspects. First, our study used data from the year 2021 and not 2020 with a different dominant variant. Second, it should be noted that the study period did not include the months of December, January and February, where the seasonality curve is found to peak, due to the restriction to two variants. Including them could lead to different estimates, but would require the consideration of at least one additional variant. The latter aspect could also contribute to the shifted curve in Thuringia, together with the observation that the infection dynamics of the Delta variant was qualitatively different in Thuringia compared to the other states, see Figs. 10 and 11.

### Behavioral parameters

For the behavioral parameters, we have chosen a form that was used and validated based on a German survey in Dönges et al. [30]. Adding to this study, we were able to identify behavioral parameters based on infection data from four German states.

We have identified (NPI-based) transmission factors that represent a higher reduction in transmission rates and hence reproduction number than a study by the Robert Koch Institute [20] which found the highest reduction of the R value at 65 %. However, the feasibility of a direct comparison of the reduction percentages is limited. First, we only have a rough estimate of $\beta _0$ based on survey data and an estimation of the secondary attack rate and were not able to identify this value in our model. Second, other studies have shown that the estimates of the R value can differ significantly based on the model and method to estimate it [101–103].

Despite this quantitative non-comparability, we observe that the identified parameters qualitatively reflect measured behavior. One direct observation was the behavioral transmission rate $\beta _0\beta _{\text {behavior}}(t)$ being highest during the summer months despite low case numbers, a feature that was also found in mobility data [104]. Seasonality however brings the actual transmission rate to sub-critical levels. This effect also aligns with literature [52] and is due to virus stability depending on sunlight and temperature [105, 106], but also behavioral components as outdoor meetings which happen more frequently in summer being linked with lower transmission risk [107].

A limitation of our study is that the self-protection effect is solely based on the state-level ICU occupancy. Despite ICU occupancy being a good estimate of adaptive behavior on a population level, other levels (national and local) could also contribute to the perceived risk. It was shown that the level of information can have an impact on the dynamics [108]. We opted to use only state-level ICU occupancy as an input for the self-protective behavior due to two reasons. First, we model the states separately to be able to compare them such that we do not want to build on national information. Second, we want all behavioral parameters to be identifiable which would be much more difficult or even impossible using district-level information.

We have observed a similar maximal effect of self-protection among the four states with values ranging from 67.6 (± 6.9) % to 81.9 (± 0.21) %. This level was reached at times when $\beta _0\beta _{\text {NPI}}$ was already considerably lower than $\beta _{0}$ and by model construction only this value was reduced by around 75 %, leading to lower absolute reductions attributable to self-protection. It remains unclear whether the same relative reduction would occur without measures as there are no times with high ICU occupancy, but no NPIs. Future work could focus on times and states with relatively few or no NPIs.

### Counterfactual simulations

To evaluate the effect of self-protection in the absence of NPIs, we performed counterfactual simulations. Even if the same relative reduction is applied when ICU occupancy was high and there are no NPIs, self-protection would not be sufficient to break the infection wave. More than observed self-protection would be needed for this.

On the other hand, the scenario without self-protection also produced huge infection waves. This means that also NPIs of the implemented strength would not have been sufficient to reduce case numbers during critical times. Combining these two conclusions, we deduct that only the combination of NPIs and self-protection by the population was able to reduce transmission dynamics below critical levels in Germany during the year 2021.

The exact numbers of the counterfactual simulations should be interpreted very cautiously as the counterfactual simulations rely on assumptions limiting their real-world applicability and are uncertain. For the scenario without self-protection, it is assumed that NPIs are implemented at the same strength and time as in reality despite totally different dynamics. This contradicts the fact that NPIs were implemented according to the epidemiological situation. For the scenario without NPIs, we extrapolate the fitted self-protection curves. This extrapolation means a non-validated continuation of the curve.

Nevertheless, both scenarios are mainly realistic when the dynamics of the counterfactuals exceeds the real dynamics. The conclusion that a single mechanism alone was not sufficient to reduce transmission dynamics to sub-critical levels is however based on their dynamics at this time. Hence, while the exact numbers are unrealistic, the main conclusion can be derived in realistic settings.

### Policy implications

The conclusion, that only the combined effect of both protection mechanisms was able to reduce the spread of COVID-19 to subcritical levels in Germany in 2021, also has important implications for policy evaluation and future public health strategies.

For policy evaluation, there are multiple studies that evaluate the effectiveness of NPIs during the Covid-19 pandemic [20–24] by the reduction in transmission. These estimates can be biased when ignoring self-protective behavior, since one would identify $\beta _{\text {NPI}}\beta _{\text {self}}$ and not only $\beta _{\text {NPI}}$. Therefore, policy evaluations should jointly model NPIs and self-protective behavior, and caution should be exercised when transferring estimated effects to other contexts where the level of self-protection may differ.

For public health strategies, policies should focus on the combination of mandated policies and the promotion of self-protective behaviors. This promotion could include information about the threat level [109], evidence on the efficacy of self-protective behavior [110–112] and facilitate self-protective behavior by providing resources like masks [113] and information about protective behaviors [114].

Although population-wide data show a correlation between ICU occupancy and self-protective behavior [2, 30], which we incorporated into our model, this relationship is unlikely to reflect a direct causal effect. This interpretation is further supported by findings that numerical variations did not significantly affect self-protective behavior [115, 116]. ICU occupancy thus appears to be a good proxy for perceived threat rather than a direct driver of behavior. For the promotion of self-protective behavior, studies investigating the underlying motivations [112, 116, 117] are therefore important. This aspect is not represented in our compartmental model.

## Conclusion

In our study, we have combined two mechanisms of contact reduction, NPIs and self-protection, into a unified modeling framework. Extending the study of Dönges et al. [30], we have identified parameters for these two mechanisms based on infection data from four German states in 2021. We have demonstrated that our model captures the key elements determining transmission dynamics effectively.

Using these parameters, we were able to show that both mechanisms had a significant impact on transmission dynamics of SARS-CoV-2 in Germany in 2021. By counterfactual simulations, which assume that one mechanism is completely absent while the other one is left unchanged, we have shown that a single mechanism alone would not have been sufficient to reduce transmission to sub-critical levels. This study stresses the importance of incorporating both mechanisms in epidemiological models, especially those used for policy evaluation.

As an example, studies aiming to estimate the impact of interventions [20–24] often neglect to account for the self-protection effect. On the other hand, epidemiological models incorporating behavioral change due to perceived risk [10, 15–17] often neglect changing NPIs. As both mechanisms can lead to high reductions in critical contacts simultaneously, modeling only one effect can lead to an overestimation of this single effect.

In summary, our results indicate that both NPIs and self-protective behavior jointly shaped transmission dynamics in Germany during 2021, and that neither mechanism alone would have sufficed to keep the wave under control. An integrated consideration of both effects is therefore essential for future modeling and policy decisions.

## Data Availability

All data used for this study is available through public repositories. They are cited in the Data description and sources section and can be found with their permanent identifiers in the reference list.

## Abbreviations

ICU: Intensive care unit
NPI: Non-pharmaceutical intervention
